# Silencing Motifs in the Clr2 Protein from Fission Yeast, *Schizosaccharomyces pombe*

**DOI:** 10.1371/journal.pone.0086948

**Published:** 2014-01-27

**Authors:** Daniel Steinhauf, Alejandro Rodriguez, Dimitrios Vlachakis, Gordon Virgo, Vladimir Maksimov, Carolina Kristell, Ida Olsson, Tomas Linder, Sophia Kossida, Erik Bongcam-Rudloff, Pernilla Bjerling

**Affiliations:** 1 Department of Medical Biochemistry and Microbiology, Science for Life Laboratory, University of Uppsala, Uppsala, Sweden; 2 Bioinformatics and Medical Informatics Laboratory, Biomedical Research Foundation of the Academy of Athens, Athens, Greece; 3 Department of Microbiology, Swedish University of Agricultural Sciences, Uppsala, Sweden; 4 Department of Animal Breeding and Genetics, SLU Global Bioinformatics Centre, Swedish University of Agricultural Sciences, Uppsala, Sweden; Duke University, United States of America

## Abstract

The fission yeast, *Schizosaccharomyces pombe*, is a well-established model for heterochromatin formation, but the exact sequence of events for initiation remains to be elucidated. The essential factors involved include RNA transcribed from repeated sequences together with the methyltransferase Clr4. In addition, histone deacetylases, like Clr3, found in the SHREC complex are also necessary for transcriptional silencing. Clr2 is another crucial factor required for heterochromatin formation found in the SHREC complex. The function of Clr2 has been difficult to establish due to the lack of conserved domains or homology to proteins of known molecular function. Using a bioinformatics approach, three conserved motifs in Clr2 were identified, which contained amino acids important for transcriptional repression. Analysis of *clr2* mutant strains revealed a major role for Clr2 in mating-type and rDNA silencing, and weaker effects on centromeric silencing. The effect on mating-type silencing showed variegation in several of the strains with mutated versions of Clr2 indicating an establishment or maintenance defect. Moreover, the critical amino acids in Clr2 were also necessary for transcriptional repression in a minimal system, by the tethering of Clr4 upstream of a reporter gene, inserted into the euchromatic part of the genome. Finally, *in silico* modeling suggested that the mutations in Clr2 cause disruption of secondary structures in the Clr2 protein. Identification of these critical amino acids in the protein provides a useful tool to explore the molecular mechanism behind the role of Clr2 in heterochromatin formation.

## Introduction

Chromatin in eukaryotic cells is an organized structure, composed of DNA together with interacting proteins. The basic unit of chromatin is the nucleosome, the nucleoprotein complex, which consists of 147 base pairs of DNA, wrapped around an octamer of four core histone proteins [1]. The histone tails protruding from the nucleosome are modified for example by acetylation and methylation. These modifications give rise to different types of chromatin with diverse properties and functions. There are two basic types of chromatin in the eukaryotic cell, heterochromatin and euchromatin. Heterochromatin was thought to be a compact transcriptionally silent structure, however it is now accepted that chromatin is flexible, and although transcription mostly occurs in the euchromatin, some transcription originates from the heterochromatic areas [2], [3]. In euchromatin there are many transcriptionally active genes as compared to heterochromatin and in addition, the latter is enriched in repetitive sequences. Heterochromatin is characterized by methylation of histone H3 lysine 9 bound by chromodomain proteins of the Heterochromatin Protein 1 (HP1) family, while euchromatin has high levels of acetylation on the histone tails and also methylation on histone H3 lysine 4 [4].

Several locations in the genome, for example the centromere, telomeres and the mating-type region in the fission yeast *Schizosaccharomyces pombe*, are heterochromatic and genes introduced into these areas are silenced, [4]–[6]. In *S. pombe*, the transcription factors that determine the mating type, P or M, are expressed from the *mat1* locus in the mating-type region. The non-expressed mating-type information is stored in the *mat2-P* or *mat3-M* cassettes, which lies in a heterochromatic area [7]. The heterochromatin in the mating-type region has several important functions; firstly, to keep the storage cassettes *mat2-P* or *mat3-M* silent; secondly, to guide the switching event in a productive way to ensure that in an M cell the *mat2-P* cassette is picked and vice versa; and thirdly, to prevent unwanted recombination events [8]. The centromeres in *S. pombe* share similarities with higher eukaryotes, such as the evolutionarily conserved CENP-A protein, a histone H3 variant that marks the site for kinetochore assembly [9]. On both sides of the central core centromeres there are inner repeats (*imr*) and outer repeats (*otr*) where the pericentromeric heterochromatin assembles [10]. Moreover, a distinct form of repressive chromatin for PolII transcribed genes is present at the rDNA repeats [11].

Heterochromatin formation in fission yeast is a multi-step process, which slightly differs for each of the various heterochromatic regions. The formation of heterochromatin at centromeres is dependent on small interfering RNAs (RNAi) transcribed from the centromeric repeats as well as on the activity of histone deacetylases, either Sir2 or Clr3 [12], [13]. This results in the recruitment of the Clr4-Rik1-Cul4 complex (ClrC), which methylates histone H3 at lysine 9, [14], [15]. Clr4-methylated H3K9 residues serve as a platform for HP1 proteins (Swi6, Chp1 and Chp2). The formation of heterochromatin in the subtelomeres and at the mating-type region on the other hand can be mediated via RNAi or other redundant mechanisms that are active in these areas [16]–[18].

The histone deacetylase Clr3 has a specific activity against histone H3K14 [19]. At least a fraction of the Clr3 proteins are part of the SHREC complex together with a Snf2 related chromatin remodeling factor, Mit1, the Zn-finger containing protein Clr1, the chromodomain protein Chp2, and Clr2, which so far has an unknown function [20], [21].

Clr2 was first identified as a factor involved in silencing of the mating-type region in *S. pombe* [22], [23]. Later on Clr2 was shown to associate with and affect, the silencing of all major heterochromatic areas in *S. pombe,* as well as the central core of the centromeres and polII transcribed genes inserted into the rDNA repeats [11], [20]. Clr2 shares no homology to other functionally annotated proteins, and lacks conserved domains of known function. However, many fungal genomes have been sequenced lately, and several of these genomes contain proteins with similarity to Clr2. Using these novel sequences a “Clr2-region”, accession number PF10383 in the Pfam database in the C-terminal half of the protein, has been established by bioinformatics analysis [24].

The role of Clr2 in heterochromatin formation and maintenance remains poorly understood. In order to get insight into the molecular function of Clr2, we used a bioinformatics approach which revealed three motifs in the Clr2 protein conserved among other fungal species. We mutated several conserved amino acids in these novel motifs and found that they were indispensable for mating-type silencing, and to a lesser extent affected transcriptional repression in the centromeric region. In addition, some of the mutated strains displayed an epigenetic switching phenotype with respect to mating-type silencing. Finally, a molecular modeling approach indicated that the introduced mutations caused disruption of the secondary structure of Clr2.

## Materials and Methods

### Bioinformatics

BLAST searches with the Clr2 protein sequence (UniProtKB O13881) were performed at the European Bioinformatics Institute (EBI) to find similar sequences in the Uniprot database [25]. Eleven UniProtKB protein sequences (B6K156, B0CTW5, D5G5I9, D1ZMD9, Q0U2S0, Q2H122, A6SNC1, D5G9P5, A7F911, B2B300, E4ZQG7) with an E-value lower than 7.0E-4 were selected to be included in an analysis using the Multiple EM for Motif Elicitation (MEME) software [26]. This analysis was done at http://meme.sdsc.edu/meme4_6_1/ using default values. Logos for the found motifs were generated using the WebLogo software [27].

### Media and strain construction

AA drop-out plates were prepared using an amino acid mix purchased from Formedium following the protocol in [28] except that adenine (58 mg/L) was added. All other media was prepared as in [29].

All the strains used in the study are listed in Table S1. Strain PJ1085, where the *clr2^+^* ORF was replaced by *ura4^+^*, was constructed using a method described in [30]. Briefly strain PJ1044 was transformed with a PCR product generated by primers D74 and D75 (Table S2) and plasmid KS-ura4 as template. Point mutations were introduced into the *clr2^+^* gene using the following strategy. The full-length *clr2^+^*gene was amplified by PCR using primers B71 and B72 (Table S2), introducing *Bam*HI sites at the 3′ and 5′ ends, using genomic DNA as template. The PCR product was then cloned into pCR®2.1-TOPO vector (Invitrogen) and sequenced. Different point mutations were introduced in *clr2^+^* by a PCR based method using Phusion (Finnzymes) [31]. The following primers were used to create the mutation: P137G F25+26, Y140G F38+F39, L142G F40+F41, R170G F27+F28, H178G F29+F30, L182G F43+F44, A375G F45+F46 and E376G F47+F48. Primers sequences are listed in Table S2. The full length and mutant *clr2* genes were sequenced and then cut out of the TOPO vector using *Bam*HI and religated into the fission yeast expression vector pREP41PkN containing three V5 (PkN) tags in the N-terminus [32]. The different versions of *V5-clr2* were amplified from the pREP41PkN (V5) vector by PCR using primers D80 and D81. The PCR products were then introduced into strain PJ1085 (*clr2::ura4^+^*) by electroporation using a Bio-Rad gene pulser (Bio-Rad) according to [33]. Cells were plated onto YEA plates and after an overnight incubation replica plated onto Fluoro-orotic acid (FOA) plates. Colonies that appeared on the FOA plates were screened by PCR for loss of the *ura4^+^* gene, using the primers A2+A3 and gain of the *V5-clr2* gene was screened using the primers A6+A7. The resulting PCR product was sequenced to confirm that only the desired mutation was introduced. Crosses were performed using standard techniques and the *V5-clr2* alleles were followed by detection of the V5 tag using primers F21+F22.

### Silencing assays and photos of yeast colonies

Spot tests were performed according to [29] using the following procedure: log-phase cultures were diluted in steps of five and drops of 5 µl were applied to the plates. The RT-qPCR was done as in [34]. Briefly, cells were allowed to grow overnight to log phase in PMG_total_ media. RNA was extracted with the RNeasy miniprep kit (Qiagen) followed by DNase treatment (Fermentas). cDNA was synthesized with the Maxima First Strand cDNA synthesis kit for RT-qPCR (Thermo scientific). RT-qPCR was then performed with Maxima SYBR green qPCR master mix (Thermo scientific) on a BioRad Mini Opticon thermo cycler (BioRad). The following primers were used: *ura4^+^* forward, CGTGGTCTCTTGCTTTTG, *ura4^+^* reverse, GTAGTCGCTTTGAAGGTTAGG; *act1^+^* forward, GGTTTCGCTGGAGATGATG, *act1^+^* reverse, ATACCACGCTTGCTTTGAG. Data is presented as *ura4^+^* transcript levels relative to *act1^+^*. Experiments were done in biological triplicate and all error bars indicate S.E.M. Canon EOS 1100D with a Canon MP-E 65 mm f/2.8 1-5X Macro Lens objective was used to take pictures of yeast colonies.

### Western analysis

*S. pombe* cells, transformed with the pREP41PkN plasmids with different versions of *V5-clr2*, resulting in over-expression of V5-Clr2 protein (PJ1279, PJ1280, PJ1440-PJ1448 in Table S1), or *S. pombe* strains with integrated *V5*-*clr2* variants at the endogenous *clr2* locus under the control of the endogenous promoter (wt and different point mutants: PJ1044-PJ1353 and PJ123-PJ1430 in Table S1) were grown in 50 ml of PMG-leu or YEA media up to the log phase (OD_600_ 0.4–0.5). Cells were collected by centrifugation at 4000 *g* for 5 min, resuspended in 3 ml of STOP buffer (450 mM NaCl, 50 mM NaF, 10 mM EDTA, 1 mM NaN_3_, pH 8.0) and aliquoted into 1.5 ml test tubes to obtain 50–70 mg of wet cell pellet upon centrifugation. Cells were resuspended in 100 ml of ice-cold RIPA buffer (50 mM Tris, pH 8.0, 150 mM NaCl, 1% Triton X-100, 0.1% SDS, 2 mM EDTA, 50 mM NaF, 0.1 mM sodium vanadate, 5 mM β-glycerophosphate, 20 mM β-mercaptoethanol), boiled for 6 min at 98°C and whole cell extracts were prepared using glass beads (400 µl of BioSpec 0.5 mm dia, Cat. No. 11079105) and FastPrep™ FP120 Cell Disrupter (max speed for 30 sec at 4°C). After beadbeating, 200 µl of Protein Extraction buffer (50 mM Tris, pH 8.0, 2.2% SDS, 2 mM EDTA, 20 mM β-mercaptoethanol) was added to each sample (to final concentration 1.5% SDS) and mixed thoroughly by vortexing. Samples were boiled for 6 min at 98°C and centrifuged at RT for 10 min at 16000 *g*. Supernatants were carefully moved into new 1.5 ml test tubes and aliquots of 2 µl were taken for protein concentration measurement, using a NanoDrop 1000 instrument. 800 µg of protein was loaded onto an 8% SDS-PAGE gel and run at 120 V for 90 min at RT. Wet transfer was performed on PVDF membrane (Immobilon-FL, Millipore) at 200 mA for 45–60 min at RT. The primary mouse anti-V5 (Invitrogen, Cat. No R960-25) and mouse anti-β-actin (Abcam, Cat. No ab8224) antibodies were used for immunoblotting with a dilution of 1∶5000 and 1∶10000, respectively. As a secondary antibody, ECL Mouse IgG HRP-linked whole Ab (GE Healthcare, Cat. No LNA931V/AG) was used with a dilution of 1∶5000. Membranes were exposed for 5–20 min using ChemiDoc™ Imaging System with Image Lab™ Software.

### Homology Modeling, Energy Minimization and Molecular Dynamics Simulations

The homology modeling for the three silencing motifs (C2SM1-3) of the Clr2 protein was performed using Modeller [35]. Subsequent energy minimization was performed using the Gromacs-implemented, Charmm27 forcefield. The crystal structure of the Haloalkane Dehalogenase (PDB entry: 3QNM) was used for the modeling of C2SM1. Likewise the crystal structures of the Hemopexin-Like Domain Of Mmp12 and Alpha-2,3- Sialyltransferase Cst-I (PDB entries: 2JXY, 2P2V) were used as template structures for the modeling of the C2SM2. Finally, the Isocitrate Lyase (PDB entry: 3I4E) was used for the modeling of C2SM3. The sequence alignments between the target sequences of the Clr2 motifs and the template sequences revealed 90%, 86%, 62% and 75% identities respectively, which allow reliable conventional homology modeling techniques to be performed. The overall homology modeling process was divided into the following steps: First, the initial spatial constraints for the target sequence were derived from a large number of template protein structures; the target sequence was aligned to the backbone of a template structure copying the geometric coordinates of the template to the target sequence. Second, target regions where geometric constrains could not be copied from the template easily, were modeled. These regions represented either deletions or insertions with respect to the template. The third step involved loop selection and side chain packing, where a collection of independent models was obtained. Fourth, the final models were scored and ranked, after they had been stereochemically tested and evaluated with a built-in module for protein geometry error detection. Models were structurally evaluated using the Procheck utility [36]. Energy minimizations were used to remove any residual geometrical strain in each molecular system, using the Charmm forcefield as it is implemented into the Gromacs suite, version 4.5.5 [37], [38]. All Gromacs-related simulations were performed though our previously developed graphical interface [39]. An implicit Generalized Born (GB) solvation was chosen at this stage, in an attempt to speed up the energy minimization process.

Molecular systems of the Clr2 constructs were subjected to unrestrained Molecular Dynamics simulations (MDs) using the Gromacs suite, version 4.5.5 [37] [38]. MDs took place in a Simple Point Charge (SPC) water-solvated, periodic environment. Water molecules were added using the truncated octahedron box extending 7 Å from each atom and molecular systems were neutralized with counter-ions as required. For the purposes of this study all MDs were performed using the NVT ensemble in a canonical environment, at 300 K, 1 atm and a step size equal to 2 femtoseconds for a total 500 nanoseconds simulation time. An NVT ensemble requires that the Number of atoms, Volume and Temperature remain constant throughout the simulation.

## Results

### Three conserved silencing motifs in the Clr2 protein

**Figure 1:**
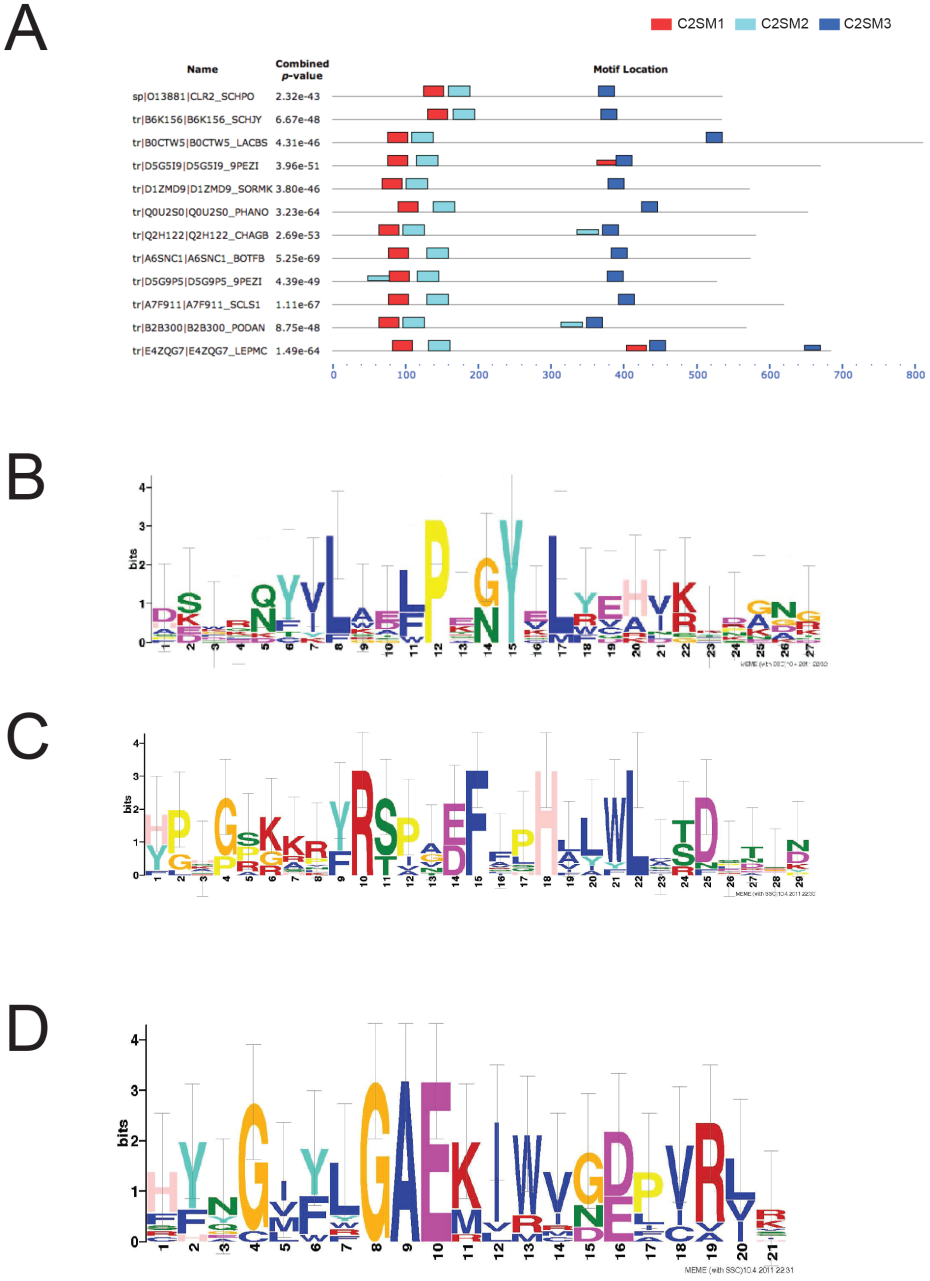
A bioinformatics approach revealed three conserved motifs in Clr2. (A) Sequence alignment between Clr2 and 11 other similar fungal proteins. Three conserved motifs, named C2SM1-3, always present in the same order were identified. C2SM1 (red) and C2SM2 (light blue) are present in the N-terminal part of Clr2 and C2SM3 (dark blue) in the C-terminal part. Height of the boxes reflects significance (*p*-value). Logos for the three motifs that highlight conserved residues (B) C2SM1 (C) C2SM2 (D) C2SM3

To investigate the molecular mechanisms of transcriptional silencing we focused on one of the least understood components in this pathway, namely Clr2. Since several fungal genomes have been sequenced lately, a similarity search among every known sequence present in the TREMBL and Swissprot sections of the InterproKB database were performed. This resulted in 50 hits when searching with the full-length Clr2 protein, none with a characterized function. Among these 50 hits, the 11 with the highest score, all of them fungal proteins, were chosen for further analysis. The top hit was from *Schizosaccharomyces japonicus* (B6K156), *S. pombes* closest relative, and the only yeast species among the 11 top hits. Moreover, all the top hits were from Ascomycota, with one exception (BOCTW5) found in a species from the Basidiomycota. A motif search was performed, using the program MEME that resulted in the identification of three conserved motifs in the Clr2 protein. Motifs 1 and 2, located in close proximity at the N-terminal of the protein, were novel and the C-terminal Motif 3 corresponded to the previously described Clr2 domain (REF IPR018839) (Figure 1A). The motifs were named Clr2 Silencing Motifs 1–3 (C2SM1-3) since they proved to be essential for transcriptional silencing (see below). C2SM1 was 27 amino acids long and at four positions displayed high levels of conservation, Leu133, Pro137, Tyr140 and Leu142 (Figure 1B). The second motif, C2SM2, had a similar size of 29 amino acids and showed 100% conservation between the 12 species at four evenly spaced amino acid positions, Arg170, Phe175, His178 and Leu182 (Figure 1C). Finally, for C2SM3 all twelve sequences shared the Gly374, Ala375 and Glu376 amino acids in a central position of the motif (Figure 1D). These conserved amino acids were used to design the mutated versions of Clr2 presented below.

### Point mutations in the Clr2 protein alleviated silencing

**Figure 2:**
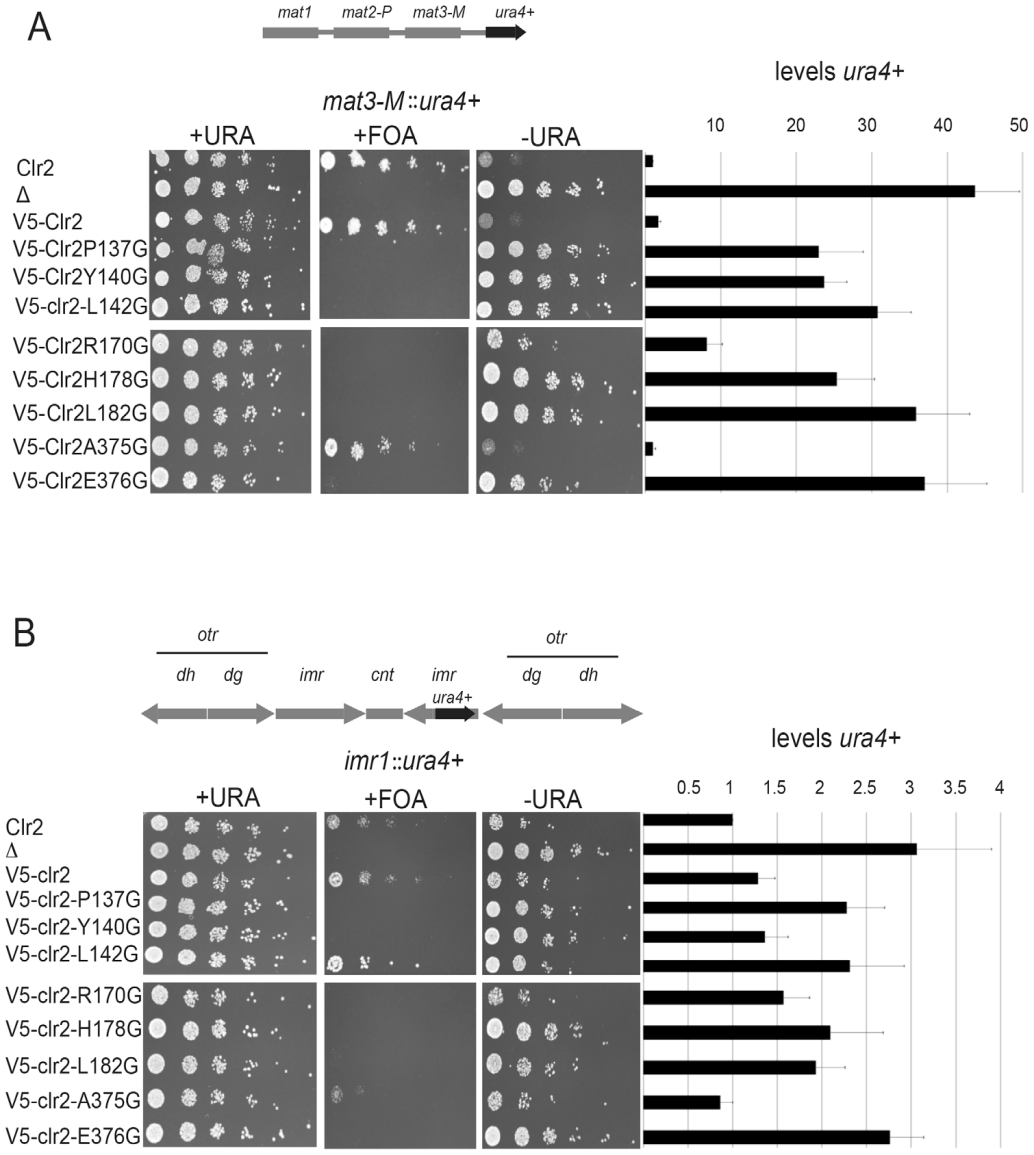
Point mutations in Clr2 resulted in derepression of reporter genes in the mating type and pericentromeric regions. Cells were serially diluted in steps of five and 5 µl were spotted onto selective media. Media used were (+URA), counter-selective (FOA) and selective (-URA) plates. The amount of *ura4^+^* transcript was quantified using RT-qPCR and the wild type expression was set to 1. Top lane is the wild type strain (Clr2) that grew poorly on the selective plates. Second lane is a strain lacking Clr2 (Δ), third contains the V5-Clr2 followed by strains expressing mutated versions of the Clr2 protein: V5-Clr2P137G, V5-Clr2Y140G, V5-Clr2L142G, V5-Clr2R170G, V5-Clr2H178G, V5-Clr2L182G, V5-Clr2A375G and V5-Clr2E376G. (A) The spotted strain carries the *mat3-M::ura4^+^* reporter in the mating type region. (B) The strains have the pericentromeric reporter gene *imr*(*Nco*I)*::ura4^+^*.

By PCR mutagenesis of *clr2*, three of the conserved amino acids in C2SM1 [proline 137 (P137), tyrosine 140 (Y140), leucine 142 (L142)], three in C2SM2 [arginine 170 (R170), histidine 178 (H178), leucine 182 (L182)], and two in C2SM3 [alanine 375 (A375) and glutamic acid 376 (E376)], were mutated. Each of the amino acids was converted to glycine, a simple amino acid with hydrogen as the variable group. The wild type *clr2^+^* gene fused to the V5 tag (*V5-clr2*), along with the mutated *clr2* bearing point mutations, also fused to the V5 tag, were introduced at the genomic *clr2* locus under the control of the endogenous promoter in strain PJ1085. In addition, this strain had an *ade6^+^* reporter gene inserted next to the *mat3-M* cassette in the normally silent mating-type region to monitor the effect of the introduced mutations on silencing [40]. The wild type, *clr2^+^*, strain grew poorly on plates lacking adenine as expected (Figure S1, first lane). It was clear that in the strain used for integration of the *V5-clr2* constructs where the *clr2* ORF had been replaced by the *ura4^+^* gene, silencing of the *mat3-M::ade6^+^* reporter gene was alleviated, visualized by growth on plates lacking adenine (Figure S1, second lane). Moreover, it was evident that the V5 tag did not prevent the Clr2 protein from silencing the *mat3-M::ade6^+^* reporter gene, since the strain with the *V5-clr2* construct integrated grew as poorly as the wild type on plates lacking adenine (Figure S1, third lane). All of the introduced mutations, except, R170G and A375G, also resulted in growth on plates lacking adenine, indicative of failure of heterochromatin to form at the mating-type region. The wild type phenotype of Clr2A376G is not surprising since a change from alanine to glycine brings little difference in amino acid properties. To be more confident in the effect of the mutations we also combined each of them with another reporter gene, *ura4^+^*, which was introduced at the same position in the mating-type region as the *ade6^+^* reporter. Moreover, the *ura4^+^* reporter has the advantage that expression can be counter-selected on FOA plates, since increased expression of the *ura4^+^* reporter results in less growth on FOA plates. The effect of the *ura4^+^* reporter resembled the growth pattern obtained with the *ade6^+^* gene (Figure 2A). In this spotting assay we also included the previously published strain, Hu582, where *his7^+^* was used to replace the entire *clr2* ORF resulting in derepression of the *ura4^+^* reporter gene inserted into the mating-type region (Figure 2A, second lane) [11]. Using the *mat3-M::ura4^+^* reporter, silencing was disrupted also in the strain carrying the R170G mutation. The degree of derepression was measured by quantifying the amount of *ura4^+^* transcripts by RT-qPCR and the results concurred well with the spotting assay, with tight repression in the wild type strain and the strains expressing V5-Clr2 and Clr2A375G while the strains with the other point mutations were derepressed to varying degree. The strain completely lacking Clr2 displayed a strong effect on *ura4^+^* transcript, since it was elevated 43 times. Mutating L182 and E376 to glycines gave the strongest effect on mating-type silencing with a 37 times increase in *ura4^+^* transcripts, while R170G was the point mutant that had the least expression of *ura4^+^*, with 8 times increase (Figure 2A).

To further characterize the mutations, *V5-clr2* together with all the mutated versions of *clr2* was combined with the *ura4^+^* reporter gene inserted into the pericentromeric heterochromatin. Serial dilutions of cells were spotted onto plates with or without uracil and onto FOA media. In this spotting assay we also included the strain PJ42, were the *clr2* gene has been replaced by *his7^+^* combined with the *ura4^+^* reporter gene inserted into the *imr* locus of centromere 1 [11]. This strain grew well on plates lacking uracil, but not at all on plates containing FOA (Figure 2B, second lane) [11]. When the effect of silencing was quantified using RT-qPCR it revealed a weak derepression since the amount of *ura4^+^* transcripts only increases 3 times in the *clr2Δ* strain as compared to the wild type strain (Figure 2B, second lane). Strains with the point-mutated versions of Clr2 affected pericentromeric silencing, also here the R170G mutation together with the Y140G mutation gave the weakest effect of around 1.5 times the wild type expression while E376G had the strongest influence since it gave 2.7 times increase in the *ura4^+^* transcription. These strains could grow on plates lacking uracil, and most of them were unable to grow on the FOA plates, similar to the complete knockout. The exception was Clr2-L142G that grew well on FOA plates as well as on plates lacking uracil, probably reflecting an unstable silencing phenotype also in the pericentromeric region for this mutant strain (Figure 2B, lane 6).

To summarize, the analysis of the *clr2* mutant strains revealed a major role for Clr2 in mating-type silencing, while the pericentromeric silencing was less dependent on Clr2.

### Variegating phenotypes in the Clr2 mutant strains

**Figure 3:**
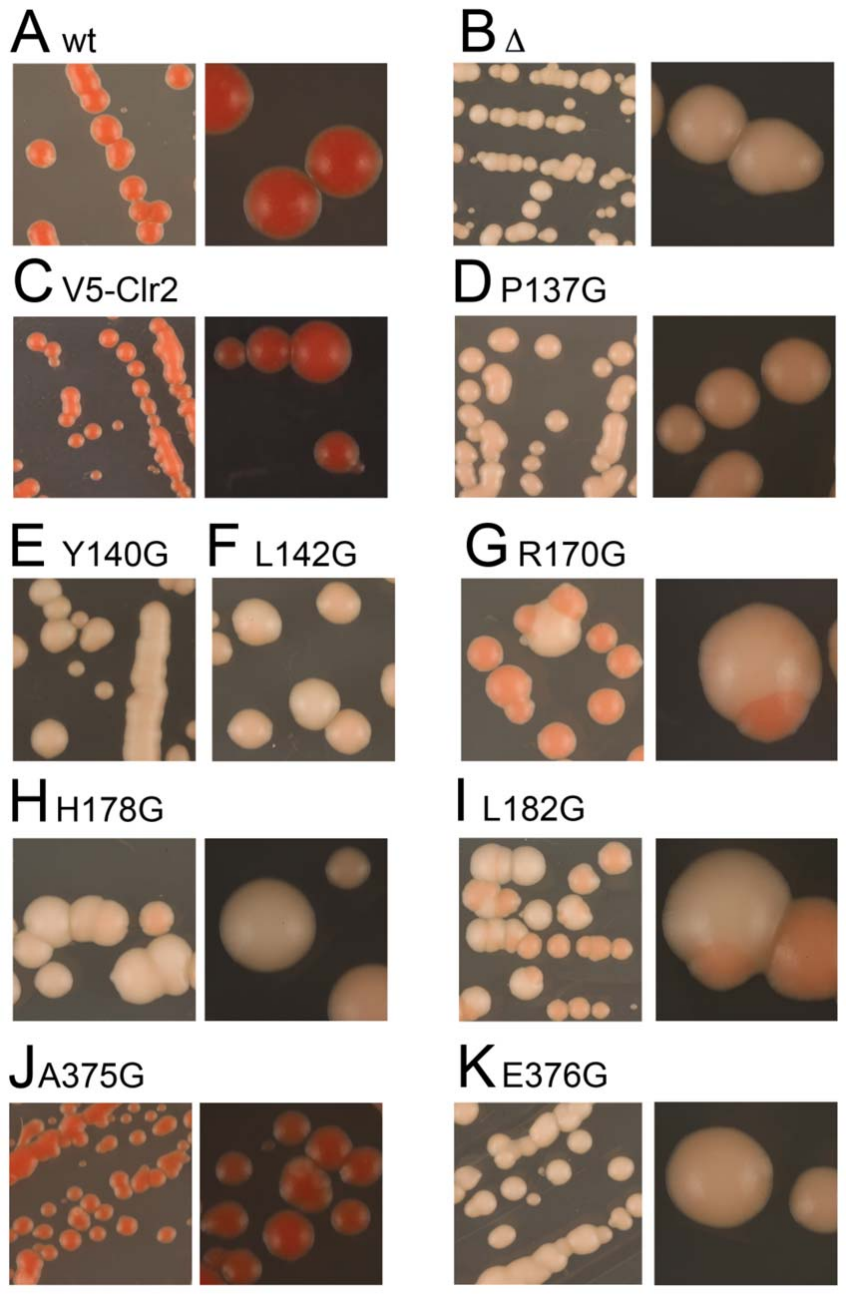
Strains containing the reporter gene *mat3-M::ade6^+^* and point mutations in Clr2 have red/white sectored colonies. All the strains carried the *mat3-M::ade6^+^*. (A) PJ1044, the wild-type strain (Clr2), (B) PJ1085, lacking Clr2 (Δ), (C) PJ1335 contains the V5-Clr2, (D) PJ1349 with V5-Clr2P137G, (E) PJ1425 with V5-Clr2Y140G, (F) PJ1424 with V5-Clr2L142G, (G) PJ1347 with V5-Clr2R170G, (H) PJ1361 with V5-Clr2H178G, (I) PJ1362 with V5-Clr2L182G, (J) PJ1363 with V5-Clr2A375G and (K) PJ1353 V5-Clr2E376G.

**Figure 4:**
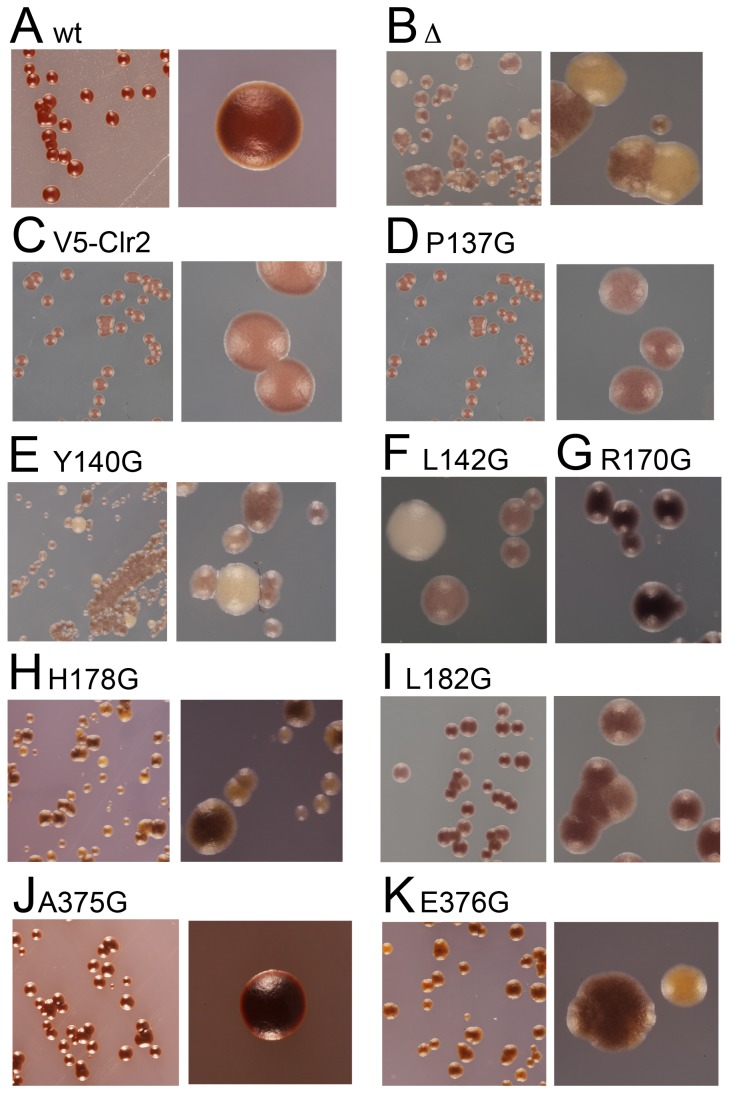
Strains containing point mutations in Clr2 has mottled and sectored iodine stained yeast colonies. (A) PJ1044, the wild-type strain (Clr2), (B) PJ1085, lacking Clr2 (Δ), (C) PJ1335 contains the V5-Clr2, (D) PJ1349 with V5-Clr2P137G, (E) PJ1425 with V5-Clr2Y140G, (F) PJ1424 with V5-Clr2L142G, (G) PJ1347 with V5-Clr2R170G, (H) PJ1361 with V5-Clr2H178G, (I) PJ1362 with V5-Clr2L182G, (J) PJ1363 with V5-Clr2A375G and (K) PJ1353 V5-Clr2E376G.

When silencing is measured with spotting assays or with RT-qPCR it is difficult to distinguish between an overall increased expression in all cells versus a situation where there are two populations of cells, one that is transcriptionally off and another that is on. To further characterize the silencing effects, we streaked the wild type strain as well as the strains carrying mutations in *clr2* on plates with low amounts of adenine, YE plates. A red pigment will accumulate in cells with low levels of Ade6 protein, resulting in red colonies when transcription is repressed (off state) and white colonies will be formed when *ade6^+^* is derepressed (on state). The wild type strain and the strain with V5-Clr2 formed exclusively red colonies, while the strain lacking Clr2 formed only white/slightly pinkish colonies (Figure 3A and B). Interestingly, the strains carrying different point mutations in Clr2 displayed different phenotypes on plates with low adenine. Part of the mutated strains had a phenotype similar to the strain completely lacking Clr2 forming white/slightly pink colonies, with the exception of L142G, R170G, L182G and A375G. The strain with the A375G mutation resembled the wild type strain, which was expected since it behaved like the wild type strain on the spotting assays (Figure 2A and 3J). The R170G mutant strain formed mainly red colonies, around 97%, indicative of a repressed *ade6^+^* gene, but occasionally white colonies were observed (Figure 3G and Table 1). Upon replating cells from two independent red colonies, on fresh YE plates the same pattern was repeated (Table 1). Two other mutant strains, L142G and L182G, that were plated on YE plates and subsequently replated, displayed distinct switching phenotypes, forming white or pink colonies in approximately equal proportions (Figure 3F, I and Table 1). Another way to monitor derepression in the mating-type region is to stain sporulating colonies with iodine vapor. The spore ascus formed when two cells of opposite mating-type fuse and undergo meiosis is stained brown, while vegetatively growing cells are stained yellow. Wild type *h^90^* strains efficiently switch mating-type resulting in a uniform brown staining by iodine vapor (Figure 4A). Deletion of the *clr2^+^* gene affects sporulation in two different ways; firstly, the directionality of switching is affected resulting in numerous unproductive switching events and secondly, there is expression from the normally silent cassettes, resulting in haploid cells containing P and M information, which makes cells undergo haploid meiosis forming aberrant spore asci and resulting in mottled staining (Figure 4B) [11]. Staining the strains with mutations in *clr2* resulted in different staining patterns, Clr2A375G stained like wild type as expected, but also R170G and P137G had a uniform dark staining with iodine vapor (Figure 4D, G and J), while strains carrying other mutations resulted in a mottled staining pattern resembling the *clr2Δ* strain, namely; Y140G, H178G and E376G. The strains with Clr2L142G and Clr2L182G had distinct phenotypes; L142G with two types of colonies, staining either brown or yellow while L182G formed mostly dark staining colonies, with a portion of the colonies being slightly lighter and more mottled (Figure 4F and I). Taken together these assays indicate that the mutations in Clr2 have different effects on heterochromatin formation in the mating-type region. Some of the mutations, Y140G, H178G and E376G, have a phenotype similar to the strain completely lacking Clr2, with a more uniform silencing effect while others, L142G, R170G and L182G, display a stronger variegating phenotype, with the reporter being either on or off.

**Table 1:** Epigenetic switching between different expression states.

Mutation	Original colony color	Number of red colonies	Number of white colonies	Percentage of red colonies	Percentage of white colonies
L142G	White	42	46		
		51	50	49.2	50.8
	Red	163	96		
		124	40	67.8	32.2
R170G	Red	63	2		
	Red	24	1	97.0	3.0
L182G	White	76	109		
	White	59	71	42.8	57.3
	Red	66	13		
	Red	89	19	82.8	17.2
	White	61	86		
	White	49	69	41.5	58.5
	Red	60	19		
	Red	67	18	77.4	22.6

Red and white colonies were picked and replated on fresh YE plates. After 3 days at 30°C the numbers of colonies with each color was counted.

### The mutant Clr2 proteins were detected by Western blot

**Figure 5:**
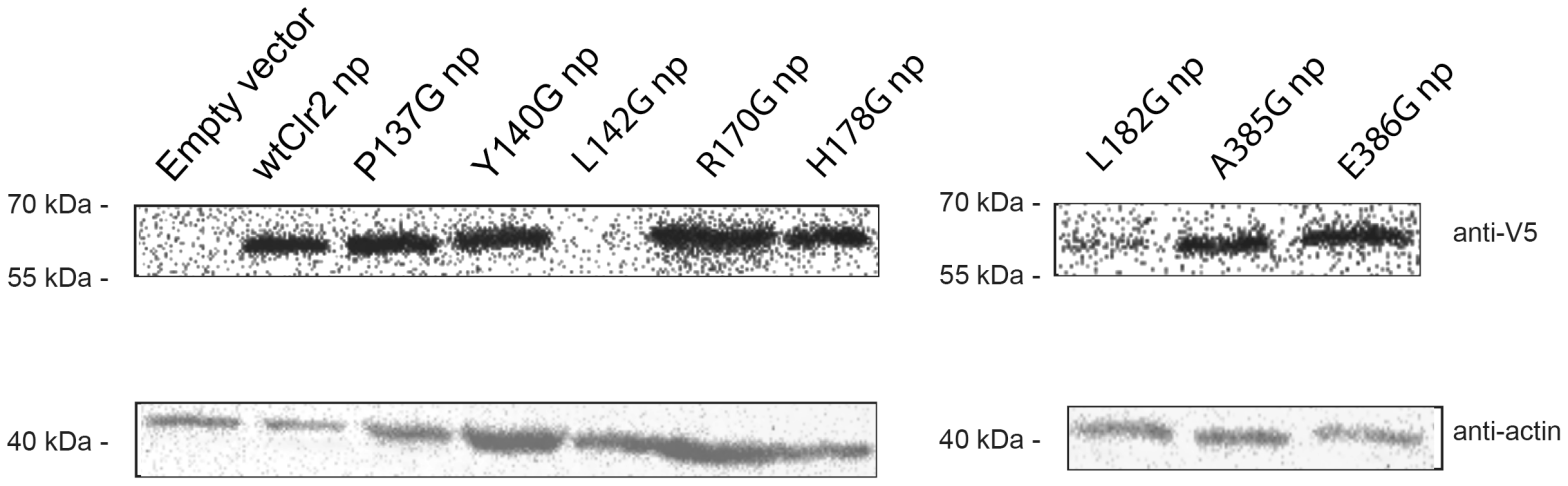
V5-Clr2 constructs were detected by Western blot. All the proteins were fused to a V5 tag in the N-terminus and detected using an anti-V5 antibody. The wild type Clr2 protein, wtClr2, as well as most of the point mutated versions of Clr2 could be detected when expressed from an *nmt* promoter (np). In all strains except L142G a protein of predicted size (63.4 kDa) was detected between the 55 and 70 kDa size markers. The mutated versions of the Clr2 protein migrated to the same position as the wild type protein and were detected by the anti-V5 antibody. Equal loading was detected using an anti-actin antibody.

To exclude the possibility that the effects on silencing were due to a mere lack of protein caused by the introduced mutations, an immunoblot analysis was performed. In order to detect the V5-Clr2 constructs the cells were transformed with the different pREP41Pk plasmids containing the various *V5-clr2* construct under the control of the medium strength *no message in thiamine* (*nmt*) promoter. This resulted in a clear detection the V5-Clr2 fusion protein with the predicted size of 63.4 kDa (Figure 5, lane 2). During over-expression the mutated versions of Clr2 were also detected, except the L142G mutant protein and a weak staining for the L182G mutant, which is possibly explained by instability of these mutant proteins (Figure 5, lane 2-8). The actin control showed equal loading of the proteins. Moreover, Western blot analysis was also performed on the strains used for the spotting assays where Clr2 was expressed from the endogenous promoter and integrated at the *clr2* locus. Most of the mutated Clr2 proteins, except P137G and L142G, could be detected but surprisingly not the wild type V5-Clr2 protein (Figure S2).

### Molecular modeling of silencing motifs in Clr2

**Figure 6:**
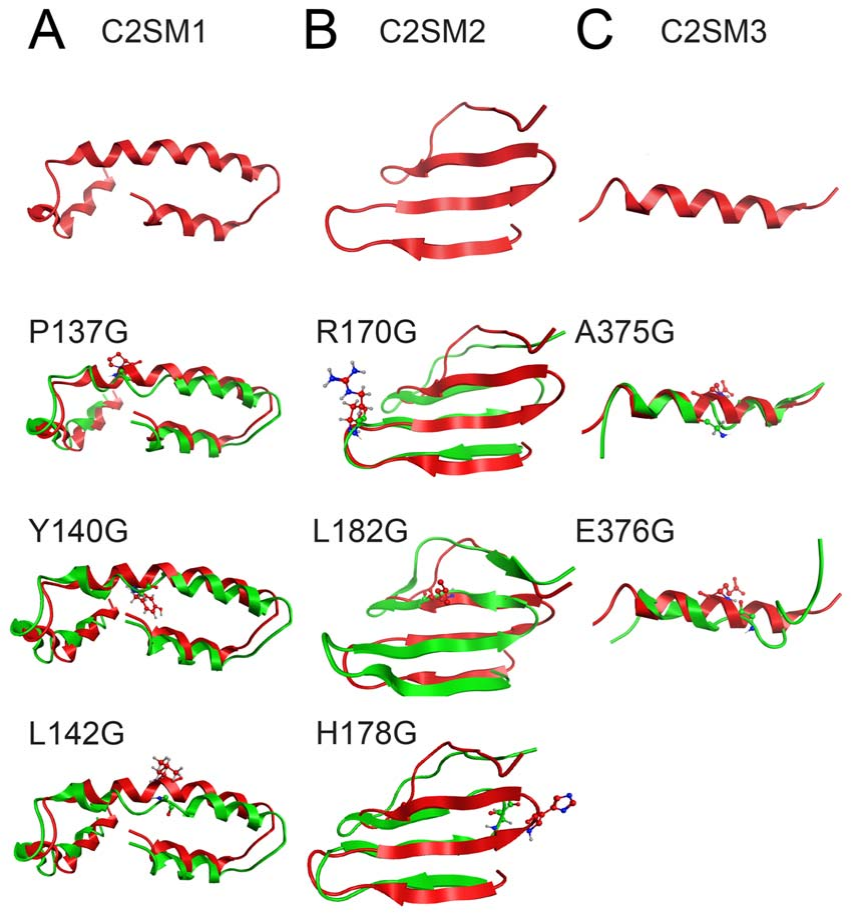
*In silico* analysis of the conserved motifs C2SM1-3 and the consequences of introducing the mutations. 3D molecular modeling study of the three conserved motifs in Clr2. C2SM1 (A), C2SM2 (B) and C2SM3 (C). Mutating the conserved amino acids in Clr2 was predicted to disrupt the secondary structure of the protein. Overlay between the predicted wild type structure in red and the simulated post-MD mutant protein conformation in green color.

Since no 3D structure information is available for the Clr2 protein we took a homology modeling approach to understand the consequences on protein folding when introducing the amino acid changes in the protein. It was not possible to generate a model of the whole Clr2 protein since not enough similarity could be found between Clr2 and any other protein with with a determined structure. However, using the Modeller software discrete parts of the Clr2 protein containing the identified motifs could be modeled (Figure 6A, B and C top panel) [35]. Moreover, the consequence of the introduced mutations could also be predicted via molecular dynamics simulations using the Gromacs software [37], [38]. Modeller predicted a α-helical conformation for C2SM 1 (Figure 6A, top). The induced P137G, Y140G and L142G mutations provoked the partial unwinding of that helix and consequent deformation of more than half of the original wild type α-helical conformation (Figure 6A and Movie S1). The wild type structure of C2SM 2 in Clr2 was predicted to consist of 3 antiparallel β-sheets (Figure 6B, top). The position of the R170 and H178 residues were modeled to be right in the middle of two of the β-sheet connecting loops (Figure 6B), while; L182 was modeled to be in the middle of one of the adjacent β-sheets. The R170G change was predicted to lead to partial loss of the first β-sheet of the three antiparallel β-sheet formations, as half of it was converted to an unstructured coil conformation (Figure 6B and Movie S2). The H178G change induced a structural loss of the second and third β-sheets of the three β-sheet formations. The L182G mutation also affected C2SM2 (Figure 6B). The hydrophobic leucine residue was predicted to actively establish hydrogen bonds to the residues in close proximity that stabilize the β-sheet structure. The L182G mutation introduces a small glycine residue in the original leucine position that was modeled to induce breakage of the contact between the two upper β-sheets (Figure 6B and Movie S3). Finally, C2SM3 was predicted to form an α-helix (Figure 6C top). The substitution of E376 to glycine in C2SM3 caused a collapse during the molecular dynamics simulation, resulting in a complete loss of the α-helical structure (Figure 6C and Movie S4). The A375G mutation was predicated to cause a partial unwinding of the α-helix secondary element. In summary, the introduced mutations in Clr2 were predicted to cause disruption of the protein's secondary structure.

### Conserved amino acids in Clr2 were necessary for silencing at several loci

**Figure 7:**
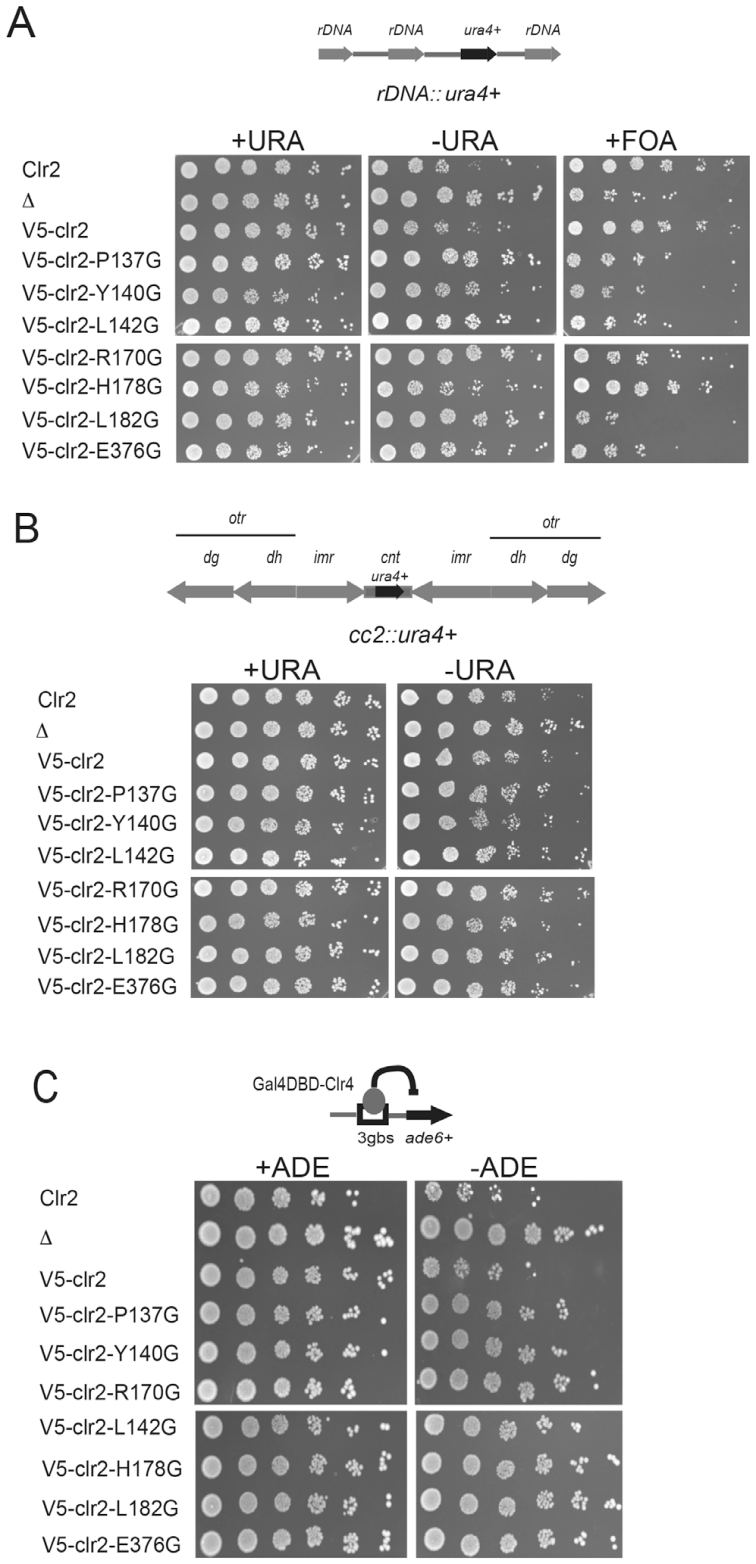
Point mutations in Clr2 affected transcriptional silencing at several locations in the *S. pombe* genome. Cells were diluted by a factor of 5 for each and spotted on +URA, selective –URA and counter-selective FOA plates. Top lane is the wild type strain (Clr2) that grows poorly on the –URA plates. Second lane is a strain lacking Clr2 (Δ), third contains the V5-Clr2 followed by the point mutations in Clr2; Clr2P137G, Clr2Y140G, Clr2L142G, Clr2R170G, Clr2H178G, Clr2L182G and Clr2E376G. The reporter gene is (A) rDNA::*ura4^+^* (B) *cc2*(*Sph*I)::*ura4^+^* and in (C) the *ade6^+^* reporter gene inserted into the *ura4* locus and with *Gal4DBD-Clr4ΔCD* resulting in repression of the *ade6^+^*gene.

To extend the investigation of how the point mutated Clr2 proteins affected transcriptional silencing, we investigated what effect the mutations had on the repression of the *ura4^+^* reporter introduced into the RNA polI transcribed genes encoding ribosomal RNA, the rDNA repeats (Figure 7A). The previously published effect of *clr2*Δ on rDNA silencing was confirmed (Figure 7A, compare lane 1 and 2) [11]. The effects of all studied mutant proteins on rDNA silencing were very similar to the complete deletion of *clr2^+^*, with the exception that the *clr2-H178G* behaved more like the wild type strain, growing well on FOA plates and poorly on plates lacking uracil. Moreover, strains were crossed to generate a combination of one of the mutations in *clr2* and a *ura4^+^* reporter gene inserted into the central core centromere 2. In this part of the genome the chromatin is composed of nucleosomes with a variant histone, CENP-A named Cnp1 in *S. pombe*, instead of the canonical histone H3. This special type of chromatin causes a weak but reproducible repression of the *ura4^+^* reporter gene that is relieved in cells lacking Clr2 (Fig. 7B, compare lane 1 and 2) [11]. The repression is so weak that even in the wild type background cells are unable to grow on the FOA plates, therefore these plates were omitted for monitoring of central core silencing. The strains with mutations in the *clr2* gene displayed weak effects on central core silencing since most of the strains grew at a comparable level to the wild type strain with the exception of L142G and R170G mutant strains that grew similar to the strain completely lacking Clr2.

The Clr4 protein, lacking its chromodomain, (Clr4ΔCD), will recruit all the factors needed for heterochromatin formation when forced to bind in a normally euchromatic area of the genome [41]. The fusion of Clr4ΔCD with Gal4 DNA binding domain (Gal4-DBD) was used to target it to Gal4 binding sites upstream of the *ade6^+^* reporter gene introduced at the *ura4* locus (*3gbs::ade6^+^*) [41]. This represents a “minimal” system to study crucial factors for formation of silent chromatin in a euchromatic environment. We tested whether the point-mutated versions of Clr2 would disrupt the ectopic silencing by Clr4ΔCD-GBD, which they did (Figure 7C). In conclusion, mutating conserved amino acids in the Clr2 protein gave the strongest effects on mating-type, rDNA and ectopic silencing and had lesser affects on centromeric silencing.

## Discussion

### Identification of three conserved motifs in the Clr2 protein

Many of the core silencing proteins in fission yeast share functional similarities with homologous proteins in humans, indicative of a strong conservation of heterochromatin formation. However, other core silencing proteins like Clr2, have no identified homologous proteins in higher eukaryotes. Since more and more genome sequences are available we searched the databases for proteins with homology to Clr2 and found many similar proteins exclusively in fungi. It is possible that Clr2 is a fungus specific protein, but we cannot exclude that there are similar proteins with functional conservation in higher eukaryotes. Three conserved motifs (C2SM1-3) were found in Clr2 when comparing 11 sequences with similarities to Clr2. The three motifs were in the same order, which underlines the conservation (Figure 1). A second search using the motifs was undertaken that identified many similar proteins, but none with a described function. There were several highly conserved amino acids in the three motifs and when these were mutated to glycine at the endogenous *clr2^+^* locus transcriptional repression in the mating-type region was disrupted (Figure S1 and 2A).

### Stability of the point mutated versions of Clr2

When the V5-Clr2 protein was expressed from a plasmid under the control of the medium *nmt* promoter it could readily be detected, as well as most of the mutated versions of the Clr2 protein, except L142G (Figure 5). However, the V5-Clr2 protein could not be detected when expressed from its endogenous promoter (Figure S2). This was unexpected since most of the mutated versions of Clr2, with the exception of P137G and L142G, could be detected when expressed from the endogenous promoter inserted at the *clr2* locus (Figure S2). These results indicated that the introduced mutations do not simply cause instability and degradation to the protein, with the exception of L142G, which could not be detected even when over-expressed.

The 3D structure of Clr2 has not been determined. However, we could find short stretches of similarity with known 3D structures for the C2SM1-3 motifs using Modeller (Figure 6 top panel), and in combination with molecular dynamics with Gromacs the consequences of the mutations could be *in silico* simulated (Figure 6 and Movie S1–4). Modeller predicted an α-helix conformation for C2SM1, a larger α-helix, flaked by two smaller ones (Figure 6A, top) with proline 137 was found in the middle larger α-helix (Figure 6A). Even though proline has very poor α-helix-forming tendency, glycine has an even lower helix-forming propensity [42]. Hence, the substitution of the proline 137, predicted to be in the α-helix in C2SM1, with a glycine most likely induces a kink to the helix, which eventually leads to unwinding and the subsequent loss of 3D structure (Figure 6A and Movie S1). The same argument applies to the Y140G and L142G mutants. Both bulkier Y140 and L142 are capable of adopting an α-helical conformation, whereas the smaller glycine substitutions are predicted to disrupt the α-helix and lead to a local complete loss of structure. Modeller predicted C2SM2 to form three antiparallel β-sheets (Figure 6B, top). The arginine 170 was predicted to be outside of the β-sheet formation most likely because there is no space available to accommodate it. However, the tiny side chain of the glycine residue substitution was small enough to allow the loop to further bend backwards towards the β-sheet formation resulting in the conversion of the first β-sheet to a coil structure (Figure 6B and Movie S2). The L182 in C2SM2 was predicted to stabilize the β-sheet formation, since the hydrophobic leucine residue actively establishes hydrogen bonds to the residues in close proximity. The introduction of a small glycine residue in the original leucine position leads to breakage of the β-sheet, which moves away from the other two β-sheets (Figure 6B and Movie S3). The H178 residue via its bulkier imidazole ring protrudes towards the outer space of the adjacent β-sheet formation, thus stabilizing the later in an optimal antiparallel conformation. However, its substitution with the smaller glycine residue (H178G) triggers a dramatic bend of the coil segment between the two β-sheets, which eventually leads to an extended loss of structure in the two β-sheet formations. Likewise the effect of mutating the glutamic acid 376 in Motif 3 is quite similar to the histidine 178 mutation. Introduction of the E376G mutation substitutes the negatively charged glutamic acid residue with an uncharged amino acid, which results in a collapse of the α-helix during the molecular dynamics simulation (Figure 6C and Movie S4). Overall, the computer simulations predicted that the point mutations in Clr2 would disrupt the secondary structures, but since most of the mutant proteins can still be detected this does not seem to lead to any major instability or loss of the proteins.

### Clr2 has its major function in mating-type, rDNA and ectopic silencing

Heterochromatin is formed at several locations in the *S. pombe* genome and the different regions have shared but also unique features. The Clr2 protein is one of the factors that contributes to transcriptional repression in all regions. Strains lacking Clr2 express reporter genes inserted into the mating-type region, pericentromeric region, central core centromere and the rDNA repeats [11]. Examples of differences between heterochromatin in the mating-type region as compared to the pericentromeric area, are that redundant factors contribute to the initiation of heterochromatin formation in the mating-type region, and that the mating-type region has a stronger silencing of inserted reporter genes (compare the first lanes in Figure 2A and B). Eight conserved amino acids were mutated in the Clr2 protein and seven of these displayed silencing defects in the mating-type, pericentromeric and rDNA regions as well as in an ectopic silencing system (Figure 2 and 7). The effect on mating-type silencing was pronounced, with around 43 times difference in expression level between the wild type and the *clr2Δ* strain (Figure 2A, lane 2). The derepression effect of the point mutations in Clr2 varied between 8 to 37 times the wild type expression level, indicating a partial silencing effect in the most of the mutant strains (Figure 2A). The strain with a *ura4^+^* reporter gene in the pericentromere displayed a weak effect on silencing when Clr2 was lacking and the strains with the point mutations had slightly weaker effects, varying between 1.3 and 2.7 (Figure 2B). The amino acid in the Clr2 protein that stood out was glutamic acid 376 since mutating this amino acid resulted in a phenotype that was almost as severe as the for the strain that completely lacked Clr2. Furthermore, the 3 times increase in *ura4^+^* transcript detected in the strain lacking Clr2 corresponded well to the previous quantification done on *clr2* mutant strains (Figure 2B) [5], [21]. The weak increase in *ura4^+^*expression can be compared to the 12 times increase in a strain lacking the methyltransferase Clr4 where the heterochromatin is completely absent, making it clear that Clr2 only marginally contributed to pericentromeric silencing [21]. The weak silencing that operates in the central core centromere was disrupted in the *clr2* knockout strain and in two of the strains carrying mutations in Clr2, namely L142G and R170G (Figure 7B). Moreover, several of the mutations in Clr2, for example *clr2-R170G*, resulted in an unstable silencing of the *ade6^+^* reporter gene in the mating-type region. This on/off epigenetic switch could be monitored as mostly red colonies but also a few white colonies on plates with limited amounts of adenine (Figure 3G and Table 1). The red and white colonies indicate that the reporter gene was most of the time turned off, presumably due to the formation of heterochromatin, but occasionally the establishment or maintenance of this chromatin state failed resulting in expression of the *ade6^+^* gene visible as white colonies. Interestingly, the R170G mutation, with the weakest *in vivo* phenotype, was *in silico* predicted to give the least drastic consequence for the Clr2 protein. It was modeled to be in an unstructured connecting loop between two β-sheets and introducing the R170G mutation was simulated to cause only partial unwinding of the first β-sheet. Another point mutation, Clr2-L182G, resulted in white and pink rather than red, colonies on plates with limited amounts of adenine, indicating that the reporter gene was never completely turned off, but the expression levels varied somewhat (Figure 3I and Table 1).

The different phenotypes of the mutated strains provide us with an excellent tool for further analysis of the Clr2 protein in heterochromatin formation. Of particular interest will be to elucidate the indicated involvement of the Clr2 protein in heterochromatin establishment or maintenance. Perhaps this issue can be further clarified by understanding the molecular role of Clr2 in the SHREC complex, for example, whether Clr2 is needed to bring in the rest of the SHREC complex, needed for the Clr3 HDAC activity, or whether Clr2 has another function?

## Conclusion

In summary, we have identified conserved silencing motifs in the Clr2 protein with critical amino acids necessary for silencing in various regions of the *S. pombe* genome. Several of the strains with mutated versions of Clr2 displayed unstable silencing phenotypes indicating deficiencies in either establishment or maintenance of heterochromatin. These mutated proteins provide us with the necessary tool to elucidate the molecular mechanism of Clr2 in heterochromatin formation.

## Supporting Information

Figure S1 **Point mutations in Clr2 resulted in derepression of reporter genes in the mating type region.** All the strains contained the *mat3-M::ade6^+^* reporter gene. Cells were serially diluted in steps of five and 5 µl were spotted onto unselective media (+ADE) and selective (-ADE) plates.
(EPS)

Figure S2 **V5-Clr2 constructs expressed from endogenous promoter were detected by Western blot.** Wild type Clr2 protein fused to a V5 tag was not detectable under the endogenous promoter, while most of the Clr2 proteins with a point mutation, except P137G and L142G were. Proteins were detected between the 55 and 70 kDa size markers consistent with the predicted size of around 63 kDa using an anti-V5 antibody. Equal loading was ensured using an anti-actin antibody. (A) Strains with the *imr1R::ura4^+^* reporter gene. (B) Strains with the *mat3-M::ade6^+^* reporter gene.
(EPS)

Movie S1 The consequence for the Clr2 proteins secondary structure by the introduction of the P137G mutation was predicted via molecular dynamics simulations.
(AVI)

Movie S2 The consequence for the Clr2 proteins secondary structure by the introduction of the R170G mutation was predicted via molecular dynamics simulations.
(AVI)

Movie S3 The consequence for the Clr2 proteins secondary structure by the introduction of the L182G mutation was predicted via molecular dynamics simulations.
(AVI)

Movie S4 The consequence for the Clr2 proteins secondary structure by the introduction of the E376G mutation was predicted via molecular dynamics simulations.
(AVI)

Table S1 List of *S. pombe* strains used in this study.
(DOCX)

Table S2 List of PCR-primers used in this study.
(DOCX)
