# Supplementary material for: Silencing Motifs in the Clr2 Protein from Fission Yeast, Schizosaccharomyces pombe
Source: PLoS One. 2014 Jan 27;9(1):e86948. doi: 10.1371/journal.pone.0086948 (PMC3903592; doi:10.1371/journal.pone.0086948)
Supplement: Table S1 — List of S. pombe strains used in this study. (DOCX) [file pone.0086948.s007.docx]

**Table S1.** List of *S. pombe* strains used in this study.

| Strain | Genotype | Source |
| --- | --- | --- |
| PJ1044 | *h^90^ mat3-M::ade6^+^ leu1-32 ura4-D18 ade6-DN/N* | This  study |
| PJ1085 | *h^90^ clr2::ura4^+^ mat3-M::ade6^+^ leu1-32 ura4-D18 ade6-DN/N* | This  study |
| PJ1335 | *h^90^ V5-clr2 mat3-M::ade6^+^ leu1-32 ura4-D18 ade6-DN/N* | This  study |
| PJ1349 | *h^90^ V5-clr2P137G mat3-M::ade6^+^ leu1-32 ura4-D18 ade6-DN/N* | This  study |
| PJ1425 | *h^90^ V5-clr2Y140G mat3-M::ade6^+^ leu1-32 ura4-D18 ade6-DN/N* | This  study |
| PJ1424 | *h^90^ V5-clr2L142G mat3-M::ade6^+^ leu1-32 ura4-D18 ade6-DN/N* | This  study |
| PJ1347 | *h^90^ V5-clr2R170G mat3-M::ade6^+^ leu1-32 ura4-D18 ade6-DN/N* | This  study |
| PJ1361 | *h^90^ V5-clr2H178G mat3-M::ade6^+^ leu1-32 ura4-D18 ade6-DN/N* | This study |
| PJ1362 | *h^90^ V5-clr2L182G mat3-M::ade6^+^ leu1-32 ura4-D18 ade6-DN/N* | This study |
| PJ1363 | *h^90^ V5-clr2A385G mat3-M::ade6^+^ leu1-32 ura4-D18 ade6-DN/N* | This study |
| PJ1353 | *h^90^ V5-clr2E386G mat3-M::ade6^+^ leu1-32 ura4-D18 ade6-DN/N* | This study |
| FY597 | *h^90^ mat3-M::ura4^+^ ura4-DS/E leu1-32 ade6-M210* | Allshire |
| Hu582 | *h^90^ mat3-M::ura4^+^ his7-366 clr2::his7^+^ ura4-DS/E leu1-32 ade6-M210* | Bjerling  2004 |
| PJ1396 | *h^90^ V5-clr2 mat3-M::ura4^+^ ura4-DS/E leu1-32 ade6-M210//DN/N* | This study |
| PJ1399 | *h^90^ V5-clr2P137G mat3-M::ura4^+^ ura4-DS/E leu1-32 ade6-M210//DN/N* | This study |
| PJ1467 | *h^90^ V5-clr2Y140G mat3-M::ura4^+^ leu1-32 ura4-DS/E ade6-M210//DN/N* | This study |
| PJ1465 | *h^90^ V5-clr2L142G mat3-M::ura4^+^ leu1-32 ura4-DS/E ade6-M210//DN/N* | This study |
| PJ1397 | *h^90^ V5-clr2R170G mat3-M::ura4^+^ ura4-DS/E leu1-32 ade6-M210//DN/N* | This study |
| PJ1451 | *h^90^ V5-clr2H178G mat3-M::ura4^+^ leu1-32 ura4-DS/E ade6-M210//DN/N* | This study |
| PJ1455 | *h^90^ V5-clr2L182G mat3-M::ura4^+^ leu1-32 ura4-DS/E ade6-M210//DN/N* | This study |
| PJ1484 | *h^90^ V5-clr2A375G mat3-M::ura4^+^ leu1-32 ura4-DS/E ade6-M210//DN/N* | This study |
| PJ1428 | *h^90^ V5-clr2E386G mat3-M::ura4^+^ ura4-DS/E leu1-32 ade6-M210//DN/N* | This study |
| PJ1279 | *h^+^ clr2::his7^+^ his7-366 cc2(SphI)::ura4^+^ leu1-32 ura4-DS/E ade6-M210*  with plasmid pREP41PkN | This study |
| PJ1280 | *h^+^ clr2::his7^+^ his7-366 cc2(SphI)::ura4^+^ leu1-32 ura4-DS/E ade6-M210*  with plasmid pREP41PkNclr2 | This study |
| PJ1446 | *h^+^ clr2::his7^+^ his7-366 cc2(SphI)::ura4^+^ leu1-32 ura4-DS/E ade6-M210*  with plasmid pREP41PkNclr2-P137G | This study |
| PJ1447 | *h^+^ clr2::his7^+^ his7-366 cc2(SphI)::ura4^+^ leu1-32 ura4-DS/E ade6-M210*  with plasmid pREP41PkNclr2-Y140G | This study |
| PJ1448 | *h^+^ clr2::his7^+^ his7-366 cc2(SphI)::ura4^+^ leu1-32 ura4-DS/E ade6-M210*  with plasmid pREP41PkNclr2-L142G | This study |
| PJ1440 | *h^+^ clr2::his7^+^ his7-366 cc2(SphI)::ura4^+^ leu1-32 ura4-DS/E ade6-M210*  with plasmid pREP41PkNclr2-R170G | This study |
| PJ1441 | *h^+^ clr2::his7^+^ his7-366 cc2(SphI)::ura4^+^ leu1-32 ura4-DS/E ade6-M210*  with plasmid pREP41PkNclr2-H178G | This study |
| PJ1443 | *h^+^ clr2::his7^+^ his7-366 cc2(SphI)::ura4^+^ leu1-32 ura4-DS/E ade6-M210*  with plasmid pREP41PkNclr2-L182G | This study |
| PJ1444 | *h^+^ clr2::his7^+^ his7-366 cc2(SphI)::ura4^+^ leu1-32 ura4-DS/E ade6-M210*  with plasmid pREP41PkNclr2-A375G | This study |
| PJ1445 | *h^+^ clr2::his7^+^ his7-366 cc2(SphI)::ura4^+^ leu1-32 ura4-DS/E ade6-M210*  with plasmid pREP41PkNclr2-E376G | This study |
| FY597 | *h^+^ imr1R(NcoI)::ura4^+^ leu1-32 ura4-DS/E ade6-M210* | Allshire |
| PJ42 | *h^+^ clr2::his7^+^ his7-366 imr1R(NcoI)::ura4^+^ leu1-32 ura4-DS/E ade6-M210//DN/N* | Bjerling  2004 |
| PJ1405 | *h^+^ V5-clr2 imr1R(NcoI)::ura4^+^ leu1-32 ura4-D18 ade6-M210//DN/N* | This study |
| PJ1407 | *h^+^ V5-clr2P137G imr1R(NcoI)::ura4^+^ leu1-32 ura4-D18 ade6-M210//DN/N* | This study |
| PJ1473 | *h^+^ V5-clr2Y140G imr(NcoI)::ura4^+^ leu1-32 ura4-DS/E ade6-M210//DN/N* | This study |
| PJ1472 | *h^+^ V5-clr2L142G imr(NcoI)::ura4^+^ leu1-32 ura4-DS/E ade6-M210//DN/N* | This study |
| PJ1406 | *h^+^ V5-clr2R170G imr1R(NcoI)::ura4^+^ leu1-32 ura4-D18 ade6-M210//DN/N* | This study |
| PJ1430 | *h^+^ V5-clr2E386G imr1R(NcoI)::ura4^+^ leu1-32 ura4-D18 ade6-M210//DN/N* | This study |
| PJ1453 | *h^+^ V5-clr2H178G imr(NcoI)::ura4^+^ leu1-32 ura4-DS/E ade6-M210//DN/N* | This study |
| PJ1459 | *h^+^ V5-clr2L182G imr(NcoI)::ura4^+^ leu1-32 ura4-DS/E ade6-M210//DN/N* | This study |
| PJ1476 | *h^+^V5-clr2A375G imr(NcoI)::ura4^+^ leu1-32 ura4-DS/E ade6-M210//DN/N* | This study |
| PJ1430 | *h^+^V5-clr2E376G imr(NcoI)::ura4^+^ leu1-32 ura4-DS/E ade6-M210//DN/N* | This study |
| Hu393 | *h^+^ rDNA::ura4^+^LEU2^+^ leu1-32 ura4-DS/E ade6-M216* | Bjerling  2004 |
| PJ34 | *h^+^ clr2::his7^+^ his7-366 rDNA::ura4^+^LEU2^+^ leu1-32 ura4-DS/E ade6-M216* | Bjerling  2004 |
| PJ1402 | *h^+^ V5-clr2 rDNA::ura4^+^LEU2^+^leu1-32 ura4-DS/E ade6-M216//DN/N* | This study |
| PJ1415 | *h^+^ V5-clr2P137G rDNA::ura4^+^LEU2^+^leu1-32 ura4-DS/E ade6-DN/N* | This study |
| PJ1537 | *h^+^ V5-clr2Y140G rDNA::ura4^+^LEU2^+^leu1-32 ura4-DS/E ade6-DN/N* | This study |
| PJ1536 | *h^+^ V5-clr2L142G rDNA::ura4^+^LEU2^+^leu1-32 ura4-DS/E ade6-DN/N* | This study |
| PJ1403 | *h^+^ V5-clr2R170G rDNA::ura4^+^LEU2^+^leu1-32 ura4-DS/E ade6-DN/N* | This study |
| PJ1535 | *h^+^ V5-clr2H178G rDNA::ura4^+^LEU2^+^leu1-32 ura4-DS/E ade6-DN/N* | This study |
| PJ1464 | *h^+^ V5-clr2L182G rDNA::ura4^+^LEU2^+^ leu1-32 ura4-DS/E ade6-M216* | This study |
| PJ1475 | *h^+^ V5-clr2E376G rDNA::ura4^+^LEU2^+^ leu1-32 ura4-DS/E ade6-DN/N* | This study |
| FY412 | *h^+^ cc2(SphI)::ura4^+^ leu1-32 ura4-DS/E ade6-M210* | This study |
| PJ32 | *h^+^ clr2::his7^+^ his7-366 cc2(SphI)::ura4^+^ leu1-32 ura4-DS/E ade6-M210* | This study |
| PJ1408 | *h^+^ V5-clr2 cc2(SphI)::ura4^+^ leu1-32 ura4-DS/E ade6-M210//DN/N* | This study |
| PJ1422 | *h^+^ V5-clr2P137G cc2(SphI)::ura4^+^ leu1-32 ura4-DS/E ade6-M210//DN/N* | This study |
| PJ1524 | *h^+^ V5-clr2Y140G cc2(SphI)::ura4^+^ leu1-32 ura4-DS/E ade6-M210//DN/N* | This study |
| PJ1525 | *h^+^ V5-clr2L142G cc2(SphI)::ura4^+^ leu1-32 ura4-DS/E ade6-M210//DN/N* | This study |
| PJ1400 | *h^+^ V5-clr2R170G cc2(SphI)::ura4^+^ leu1-32 ura4-DS/E ade6-M210//DN/N* | This study |
| PJ1523 | *h^+^ V5-clr2H178G cc2(SphI)::ura4^+^ leu1-32 ura4-DS/E ade6-M210//DN/N* | This study |
| PJ1463 | *h^+^ V5-clr2L182G cc2(SphI)::ura4^+^ leu1-32 ura4-DS/E ade6-M210* | This study |
| PJ1457 | *h^+^ V5-clr2E376G cc2(SphI)::ura4^+^ leu1-32 ura4-DS/E ade6-M210//DN/N* | This study |
| PJ1231 | *h^+^ GBD-clr4ΔCD::hphMX6 ura4::3gbs-ade6^+^ leu1-32 DN/N* | This study |
| PJ1327 | *h^+^ clr2::ura4^+^ GBD-clr4ΔCD::hphMX6 ura4::3gbs-ade6^+^ leu1-32 DN/N* | This study |
| PJ1395 | *h^+^ V5-clr2 GBD-clr4DCD::hphMX6 ura4::3gbs-ade6^+^ leu1-32 ade6-DN/N* | This study |
| PJ1369 | *h^+^ V5-clr2P137G GBD-clr4DCD::hphMX6 ura4::3gbs-ade6^+^ leu1-32 ade6-DN/N* | This  study |
| PJ1550 | *h^+^ V5-clr2Y140G GBD-clr4DCD::hphMX6 ura4::3gbs-ade6^+^ leu1-32 ade6-DN/N* | This  study |
| PJ1521 | *h^+^ V5-clr2L142G GBD-clr4DCD::hphMX6 ura4::3gbs-ade6^+^ leu1-32 ade6-DN/N* | This  study |
| PJ1367 | *h^+^ V5-clr2R170G GBD-clr4DCD::hphMX6 ura4::3gbs-ade6^+^ leu1-32 ade6-DN/N* | This  study |
| PJ1483 | *h^+^ V5-clr2L182G GBD-clr4DCD::hphMX6 ura4::3gbs-ade6^+^ leu1-32 ade6-DN/N* | This  study |
| PJ1478 | *h^+^ V5-clr2E376G GBD-clr4DCD::hphMX6 ura4::3gbs-ade6^+^ leu1-32 ade6-DN/N* | This  study |
