# Supplementary material for: Silencing Motifs in the Clr2 Protein from Fission Yeast, Schizosaccharomyces pombe
Source: PLoS One. 2014 Jan 27;9(1):e86948. doi: 10.1371/journal.pone.0086948 (PMC3903592; doi:10.1371/journal.pone.0086948)
Supplement: Table S2 — List of PCR-primers used in this study. (DOCX) [file pone.0086948.s008.docx]

**Table S2**. List of PCR-primers used in this study

| **Primer name** | **Primer sequence** |
| --- | --- |
| D74 | TCAATTTTCCGCTACACAAAATAATTTGCATACTTCTTTTGTTCCGTTATAACTTCAGCTATTGTCATTTTTAGTAGCTAATGGATGCTAGAGTATTTCA |
| D75 | ATATTATTTACATTATAAATAATGACCGTAACTGATCAATAAGTAGACACTTCTATATCTAATTTTATCTGCCATACTTATTAATGCTGAGAAAGTCTTT |
| B71 | CCCGGATCCCCATGCCTGCTATTACTTGTGTTTG |
| B72 | CCCGGATCCTTATTACATTACAACTGCTGACACC |
| D80 | TCAATTTTCCGCTACACAAAATAATTTGCATACTTCTTTTGTTCCGTTATAACTTCAGCTATTGTCATTTTTAGTAGCTAGAGCTCATGGGTATTCCTAA |
| D81 | ATATTATTTACATTATAAATAATGACCGTCACTGATCAATAAGTAGACACTTCTATATCTAATTTTATCTGCCATACTTATTACATTACAACTGCTGACA |
| F25 | GAAAATTATTATCTTGCCTCTTTAGGCACCAATTACCAG |
| F26 | CTGGTAATTGGTGCCTAAAGAGGCAAGATAATAATTTTC |
| F27 | GTGGTCGACCATTTGGATCAGTTAATGATTTC |
| F28 | GAAATCATTAACTGATCGAAATGGTCGACCAC |
| F29 | GTTAATGATTTCCTTCATGGTTTGTATTGGCTTATATC |
| F30 | GATATAAGCCAATACAAACCATGAAGGAAATCATTAAC |
| F38 | CTTGCCTCTTTACCCACCAATGGCCAGCTATATCAGCGTG |
| F39 | CACGCTGATATAGCTGGCCATTGGTGGGTAAAGAGGCAAG |
| F40 | CTTTACCCACCAATTACCAGGGATATCAGCGTGATTCAAAC- |
| F41 | GTTTGAATCACGCTGATATCCCTGGTAATTGGTGGGTAAAG |
| F43 | CATCATTTGTATTGGGGTATATCAGATTTGACCCGTAAC |
| F44 | GTTACGGGTCAAATCTGATATACCCCAATACAAATGATG |
| F45 | GAATTTTCCGTGGAGGCGAAAAACTATGGATTAATGATTTATGTG |
| F46 | CACATAAATCATTAATCCATAGTTTTTCGCCTCCACGGAAAATTC |
| F47 | GAATTTTCCGTGGAGCCGGAAAACTATGGATTAATGATTTATGTG |
| F48 | CACATAAATCATTAATCCATAGTTTTCCGGCTCCACGGAAAATTC |
| A2 | GAGGGGATGAAAATTCCCA |
| A3 | TTCGACAACAGGATTACGAC |
| A6 | TCACTGATCAATAAGTAGACAC |
| A7 | GCGTCAATTTTCCGCTACAC |
| F21 | GTAAACGACCGCATATCTG |
| F22 | ATGCCTGCTATTACTTGTG |
